# The diagnostic significance of Doppler ultrasound assessment of superior mesenteric artery hemodynamic changes in acute suppurative appendicitis: an integrated analysis of systemic inflammation and oxidative stress

**DOI:** 10.3389/fmed.2026.1806137

**Published:** 2026-03-10

**Authors:** Jiaoran Liu, Haihua Sun, Long Peng

**Affiliations:** Department of Ultrasound Diagnostic, Bethune International Peace Hospital, Shijiazhuang, China

**Keywords:** acute suppurative appendicitis, hemodynamics, inflammation, superior mesenteric artery, ultrasound

## Abstract

This study aimed to evaluate the hemodynamic changes of the superior mesenteric artery (SMA) in patients with acute suppurative appendicitis (ASA) and to explore its diagnostic value. A retrospective study was conducted on 103 patients diagnosed with ankylosing spondylitis (ASA) by ultrasound examination and pathology at our hospital between January 2015 and December 2022, who were included in the ASA group. Another 98 healthy volunteers with normal physical examination results in 2021 were selected as the control group. Hemodynamic parameters of spondylitis were measured by ultrasound, including peak systolic velocity (PSV), end-diastolic velocity (EDV), resistance index (RI), and systolic/diastolic ratio (S/D). Receiver operating characteristic (ROC) curves were plotted for statistically significant parameters, and their optimal cutoff values were determined. Compared with the control group, the PSV, RI, and S/D values of the ASA group were significantly increased, and the differences were statistically significant (all *p* < 0.05). The median PSV in the ASA group was 215.9 cm/s (interquartile range: 169.1–233.7 cm/s), while that in the control group was 132.8 cm/s (interquartile range: 118.1–155.0 cm/s). The RI in the ASA group was 0.83 (interquartile range: 0.79–0.85), while that in the control group was 0.73 (interquartile range: 0.69–0.77). The S/D ratio in the ASA group was 5.8 (interquartile range: 4.7–6.7), significantly higher than that in the control group was 3.7 (interquartile range: 3.2–4.4). ROC curve analysis showed that the optimal cutoff value for PSV was 157.5 cm/s, and its area under the curve (AUC) was 0.89. Ultrasound assessment of superior mesenteric artery hemodynamics is of significant value in the auxiliary diagnosis of acute suppurative appendicitis (ASA). Elevated SMA-PSV is closely associated with systemic inflammatory response, oxidative stress, and the expression of ferroptosis-related molecules, and can serve as a reliable indicator of the pathophysiological state of ASA. When direct ultrasound imaging of the appendix is limited, SMA hemodynamic measurement can serve as a non-invasive alternative method to aid in early diagnosis and clinical decision-making.

## 1 Introduction

Acute appendicitis (AA) is the leading cause of acute abdominal pain requiring emergency medical attention worldwide (1, 2). It is also a common condition in pediatric patients, with a higher incidence in males than females, primarily affecting individuals aged 10–30 years (3). Histologically, AA can be classified into four types: simple, suppurative, perforated, and gangrenous appendicitis (3). The exact risk factors for AA include luminal obstruction, high sugar intake, low dietary fiber consumption, and infections caused by viruses, bacteria, or parasites (4, 5).

Clinically, AA often presents with right iliac fossa pain, anorexia, nausea, constipation, and vomiting (6, 7). However, this classic presentation is observed in only about 50% of cases, making diagnosis challenging. ASA is a moderate stage of AA characterized by acute suppurative infection with rapid progression, which can lead to systemic inflammatory responses and severe complications such as perforation and sepsis, posing a significant threat to life (8). Surgical intervention remains the primary treatment for ASA, highlighting the importance of early and accurate diagnosis (9–12).

ASA diagnosis typically relies on clinical symptoms and laboratory tests. However, due to the diverse and sometimes atypical presentations of ASA, misdiagnosis and missed diagnoses are common. With advancements in ultrasound technology, gastrointestinal ultrasound has become a widely used diagnostic tool for AA (13). Despite its advantages, ultrasound diagnosis of ASA is not always definitive due to factors such as intestinal gas interference, obesity, and operator experience, which can limit its accuracy. Therefore, exploring novel diagnostic approaches for ASA is crucial to improving clinical outcomes.

The superior mesenteric artery (SMA), a major visceral branch of the abdominal aorta, supplies blood to the intestines, including the appendix through its arterial branches. Hemodynamic changes in the SMA reflect intestinal circulatory alterations and can be influenced by various gastrointestinal pathologies, including inflammatory bowel diseases such as Crohn’s disease and ulcerative colitis, which are known to cause mesenteric vascular dilation and congestion (14). Changes in SMA blood flow may also occur secondary to hemodynamic alterations in the appendiceal artery (15, 16).

Despite the growing understanding of mesenteric arterial hemodynamics in inflammatory bowel diseases, research on the diagnostic value of SMA hemodynamics in ASA remains limited. Therefore, this study aims to investigate the hemodynamic changes in the SMA of ASA patients using Doppler ultrasound and evaluate the potential of SMA hemodynamic parameters as a diagnostic tool for ASA, providing valuable clinical insights for improved diagnosis and management.

## 2 Materials and methods

### 2.1 Study design and population

This prospective study was conducted at Bethune International Peace Hospital. A total of 103 patients diagnosed with acute suppurative appendicitis (ASA) based on ultrasound findings and confirmed by pathological examination were included. These patients were admitted to our hospital between January 2015 and December 2022.

#### 2.1.1 Inclusion criteria

Patients were eligible for inclusion if they met the following criteria:

1. Diagnosis of ASA confirmed by ultrasound findings.
2. Age between 20 and 30 years.
3. Postoperative confirmation of ASA through pathological examination.

#### 2.1.2 Exclusion criteria

Patients were excluded from the study if they met any of the following conditions:

1. Diagnosis of appendiceal perforation or periappendiceal abscess based on ultrasound examination.
2. Presence of pre-existing superior mesenteric artery (SMA) disease detected by ultrasound.

Initially, 112 patients were diagnosed with ASA via ultrasound, of whom 103 were confirmed through pathological examination and included in the study. The ASA group consisted of 56 males and 47 females, with an age range of 20–30 years and a median age of 25 years (IQR: 22–28).

A control group of 98 healthy volunteers was recruited, all of whom had normal results in their annual physical examinations in 2021. None of the control participants had a history of malignancy, major surgery, chronic gastrointestinal or genitourinary disease, or chronic cardiovascular disease. The control group comprised 51 males and 47 females, with an age range of 20–30 years and a median age of 25 years (IQR: 23–28).

Body mass index (BMI) was calculated as weight (kg) divided by height squared (m^2^). SPSS 27.0 statistical software was used for data analysis.

### 2.2 Ultrasound examination and hemodynamic measurements

#### 2.2.1 Equipment and protocol

All ultrasound examinations were performed using the ALOKA SSD-α10 ultrasound system, equipped with 2–4 MHz curved-array and 5–13 MHz linear probes to optimize visualization in ASA patients.

Participants were required to fast for at least 8 h before the examination. All ultrasound scans were conducted in the supine position following a 5-min rest period to ensure consistent measurements.

Abdominal ultrasound was first performed using a curved-array probe to locate the ileocecal region and identify the appendix. If needed, a high-frequency linear probe was used for further assessment until the appendix was clearly visualized.

#### 2.2.2 Ultrasound diagnostic criteria for ASA

The ultrasound criteria for ASA diagnosis included:

Enlarged appendix with a luminal diameter >10 mm.
Marked thickening of the submucosal layers of the appendix.
Increased periappendiceal echogenicity, indicative of inflammation.

Once the diagnosis of ASA was established, an abdominal probe was used to assess the superior mesenteric artery (SMA).

#### 2.2.3 Doppler assessment of SMA

To ensure accurate hemodynamic measurements, the Doppler angle was carefully adjusted to maintain an angle of <60° between the ultrasound beam and the direction of SMA blood flow.

Sonographic measurements were obtained in the longitudinal plane, within the first 3 cm of the SMA trunk, before the emergence of its branches. The sample volume was placed in the center of the SMA lumen, approximately 0.5–1 cm from the arterial origin, ensuring that the sampling gate width was set to half the vessel diameter without contacting the vessel walls.

During the examination, patients were instructed to hold their breath momentarily to minimize respiratory motion artifacts.

#### 2.2.4 Measured hemodynamic parameters

The following SMA hemodynamic parameters were recorded:

Peak systolic velocity (PSV).
End-diastolic velocity (EDV).
Resistance index (RI).
Systolic/diastolic ratio (S/D ratio).

For each patient, three consecutive measurements were obtained, and the mean value of the three measurements was used for statistical analysis to reduce random error.

All ultrasound examinations and measurements were conducted by senior radiologists, ensuring consistency and minimizing operator-dependent variability. The same measurement protocol was applied to both the ASA and control groups.

### 2.3 RNA extraction and cDNA synthesis

Total RNA was extracted using the RNAeasy™ Blood RNA Isolation Kit (with rotating column) (Beyotime, China). The purity and concentration of the extracted RNA were determined using a NanoDrop 2000 spectrophotometer (Thermo Fisher Scientific, USA). Using 1 μg of the extracted RNA as a template, first-strand complementary DNA (cDNA) was synthesized using the BeyRT™ III First-Strand cDNA Synthesis Kit (Beyotime, China).

### 2.4 Enzyme-linked immunosorbent assay (ELISA) and blood smear

Human interleukin-6 (MDA) and tumor necrosis factor-α (MDA) assay kits were purchased from Nanjing Jiancheng Bioengineering Institute; human C-reactive protein (MDA) assay kits were purchased from Wuhan Yunclone Technology Co., Ltd.; malondialdehyde (MDA), superoxide dismutase (MDA), GPX4, and ACSL4 assay kits were purchased from Shanghai Kebo Biotechnology Co., Ltd. After the colorimetric reaction, the optical density (OD) value of the blank wells was calibrated to zero, and the OD was measured at 450 nm using an ELISA reader, with each well measured three times. In addition, leukocytes, neutrophils, and lymphocytes were counted using the blood smear method. A drop of patient blood was placed on a glass slide, which was tilted at 45° to ensure uniform blood distribution. After the slide dried, Wright staining was performed, and the staining solution completely covered the blood smear. The slide was allowed to stand for 5 min, then phosphate buffer was added, and the slide was allowed to stand for another 10 min. The slides were then rinsed with sterile double-distilled water and air-dried before being observed under a microscope. Counts were taken three times for each field of view to ensure accuracy.

### 2.5 Western blotting (WB)

Tissue proteins were extracted using RIPA lysis buffer (Beyotime Biotechnology Co., Ltd., China), with 10% protease and phosphatase inhibitors (NCM Biotechnology Co., Ltd., China) added to the lysis buffer. After protein concentration determination, 30 μg of protein from each sample was separated by SDS-PAGE: first electrophoresis at 80 V for 30 min, then at 120 V for 60 min. Subsequently, the gel protein was transferred to a PVDF membrane at 250 mA for 100 min. After transfer, the membrane was blocked with 5% skim milk at room temperature for 60 min, and then incubated with primary antibody overnight at 4 °C. The next day, the membrane was washed three times with TBST buffer and incubated with secondary antibody at room temperature for 60 min. Protein signals were detected using a ChemiDoc XRS+system (Bio-Rad, Inc., USA). The antibodies used included *β*-actin (#AF7018), 1 L-6 (#DF2844), TNF-*α* (#AF7014), Endothelin-1 (#DF6125), and eNOS (#AF0096), all of which were purchased from Proteintech (China).

### 2.6 Statistical analysis

Data visualization was performed using GraphPad Prism 9. Categorical variables were expressed as percentages (%), while continuous variables were presented as mean ± standard deviation (SD) or median (range). The *χ*^2^ test was used to analyze categorical variables. Student’s *t*-test was applied for normally distributed continuous data, whereas the Mann–Whitney U test was used for non-normally distributed variables. The diagnostic performance of PSV in ASA detection was assessed using receiver operating characteristic (ROC) curve analysis. A *p*-value <0.05 was considered statistically significant.

## 3 Results

### 3.1 Comparison of SMA hemodynamics between the ASA Group and control group

**Figure 1:**
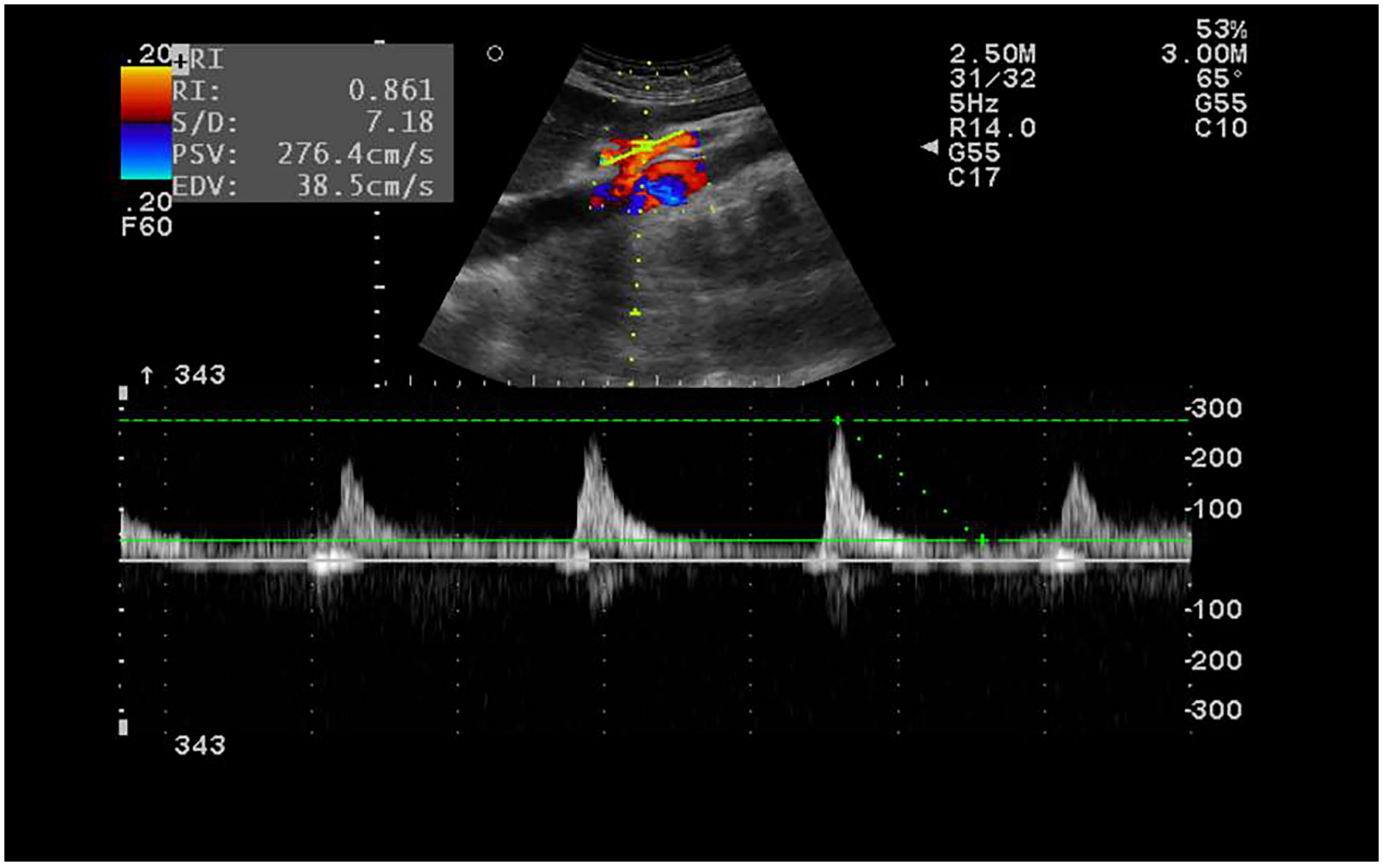
The hemodynamic test of SMA. Male, 28 years old, metastatic right lower abdominal pain 1 day examination. RI was 0.86, S/D was 7.2.

**Figure 2:**
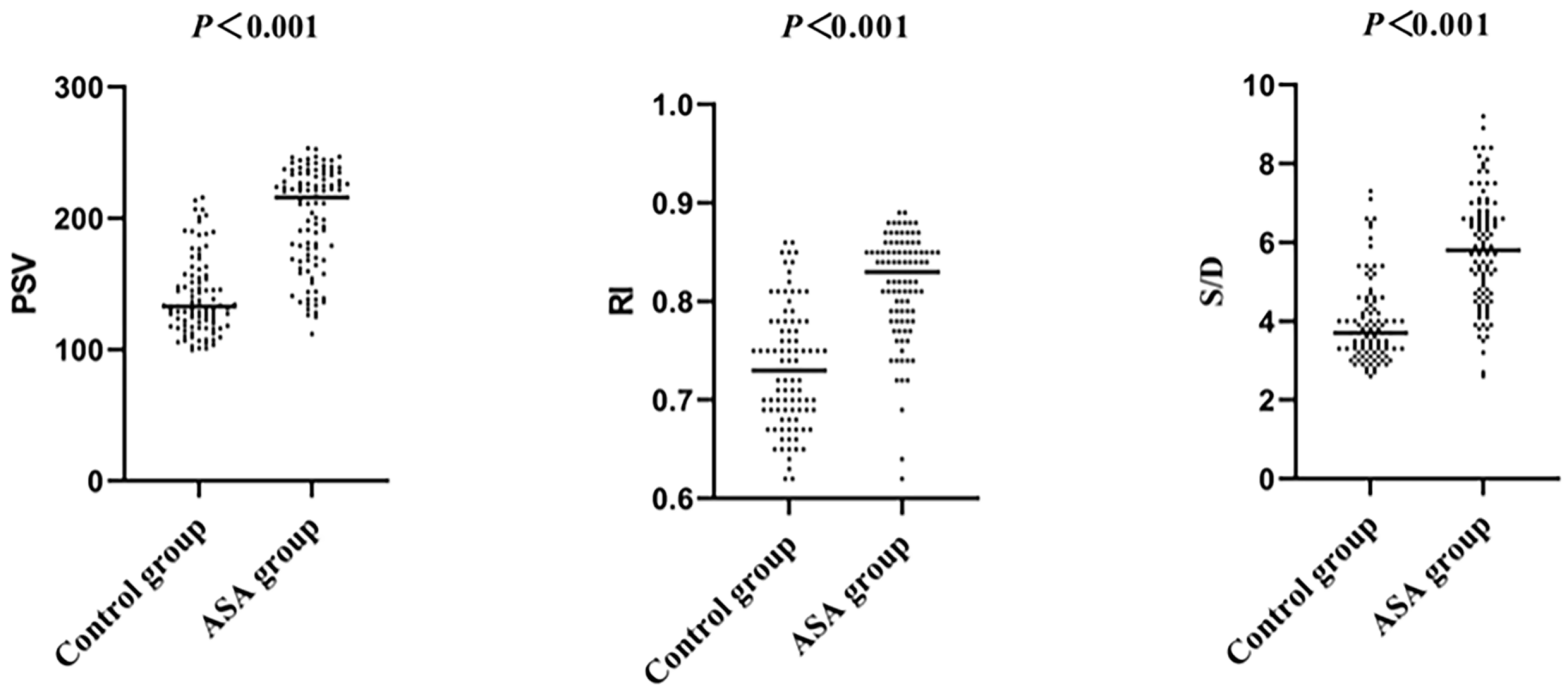
The PSV, RI, and S/D of ASA patients was significantly higher than that of the control group (*p* < 0.01).

There were no significant differences between the ASA group and the control group in terms of sex, age, and body mass index (BMI) (*p* > 0.05). Detailed demographic and clinical characteristics are shown in Table 1. Representative ultrasound images of ASA patients are shown in Figure 1. Hemodynamic parameters, including peak systolic velocity (PSV), end-diastolic velocity (EDV), resistance index (RI), and systolic/diastolic ratio (S/D ratio), were compared between the two groups. The PSV in the ASA group was 215.9 (168.9–233.7) cm/s, significantly higher than that in the control group [132.8 (118.1–155.0) cm/s] (*p* < 0.05). Similarly, the RI in the ASA group was 0.83 (0.79–0.85), significantly higher than that in the control group [0.73 (0.69–0.77)] (*p* < 0.05). Furthermore, the S/D ratio in the ASA group was 5.8 (4.7–6.7), significantly higher than that in the control group (3.7, 3.2–3.7) (*p* < 0.05). In contrast, no significant difference was observed in EDV between the two groups (*p* > 0.05). Although no significant difference was observed in end-diastolic flow velocity (EDV) between the two groups (*p* > 0.05), the hemodynamic parameters in the ASA group showed an overall trend of abnormally high values, suggesting that ASA may be closely related to hemodynamic abnormalities, thus providing hemodynamic evidence to support its clinical manifestations. Specific statistical data and graphical results can be found in Table 1 and Figures 2A–C.

**Table 1:** Baseline characteristics of two groups.

Characteristic	The control group	The ASA group	*p*
Male, %	51 (55.1)	56 (54.4)	0.78
Female, %	47 (44.9)	47 (45.6)	0.78
Age	25 (23–28)	25 (22–28)	0.70
BMI	23.0 ± 1.8	23.4 ± 2.7	0.21
PSV	132.8 (118.1–155.0)	215.9 (168.9–233.7)	<0.01
EDV	36.2 ± 5.4	35.4 ± 5.7	0.31
RI	0.73 (0.69–0.77)	0.83 (0.79–0.85)	<0.01
S/D	3.7 (3.2–4.4)	5.8 (4.7–6.7)	<0.01

Data are presented as mean ± SD and median (interquartile range).

### 3.2 The value of SMA hemodynamics in the diagnosis of ASA

**Figure 3:**
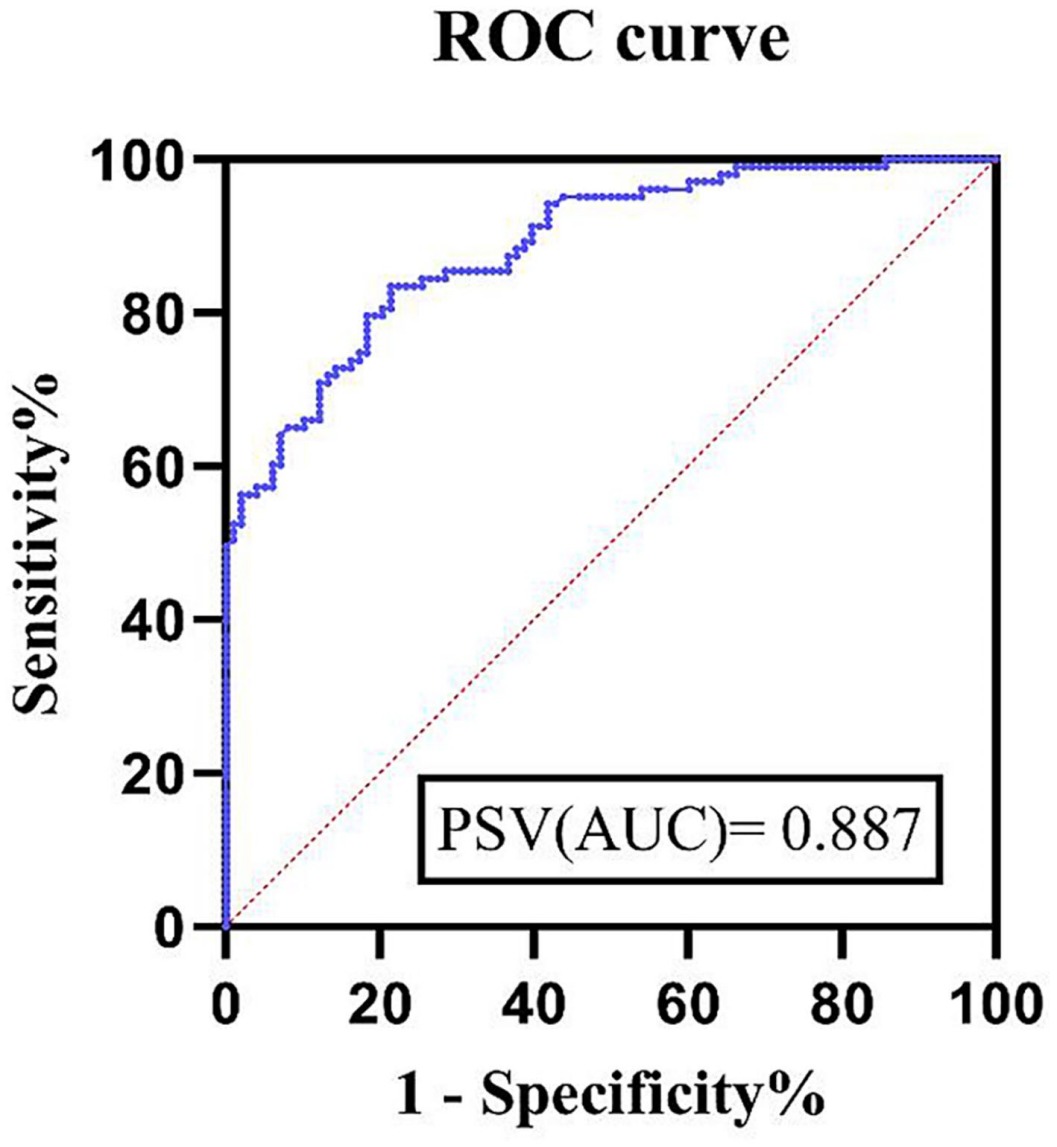
ROC curve of patients with ASA diagnosed by PSV.

To evaluate the efficacy of peak systolic velocity (PSV) of the superior mesenteric artery (SMA) in the diagnosis of acute suppurative appendicitis (ASA), receiver operating characteristic (ROC) curves were plotted (Figure 3). The results showed that the area under the curve (AUC) of PSV for diagnosing ASA was 0.89 (95% CI, 0.84–0.93, *p* < 0.01), indicating good diagnostic efficacy. The optimal cutoff value for PSV was 157.5 cm/s; a PSV above this threshold could be considered for the diagnosis of ASA. At this cutoff value, the diagnostic sensitivity was 83.50%, and the specificity was 78.57%. These results indicate that SMA hemodynamic assessment, especially the PSV parameter, has significant potential for application as a non-invasive detection method in the auxiliary diagnosis of ASA. The detailed results of the ROC curve analysis are shown in Figure 3.

### 3.3 Characteristics of changes in systemic inflammation, oxidative stress, and ferroptosis-related markers in ASA patients

**Figure 4:**
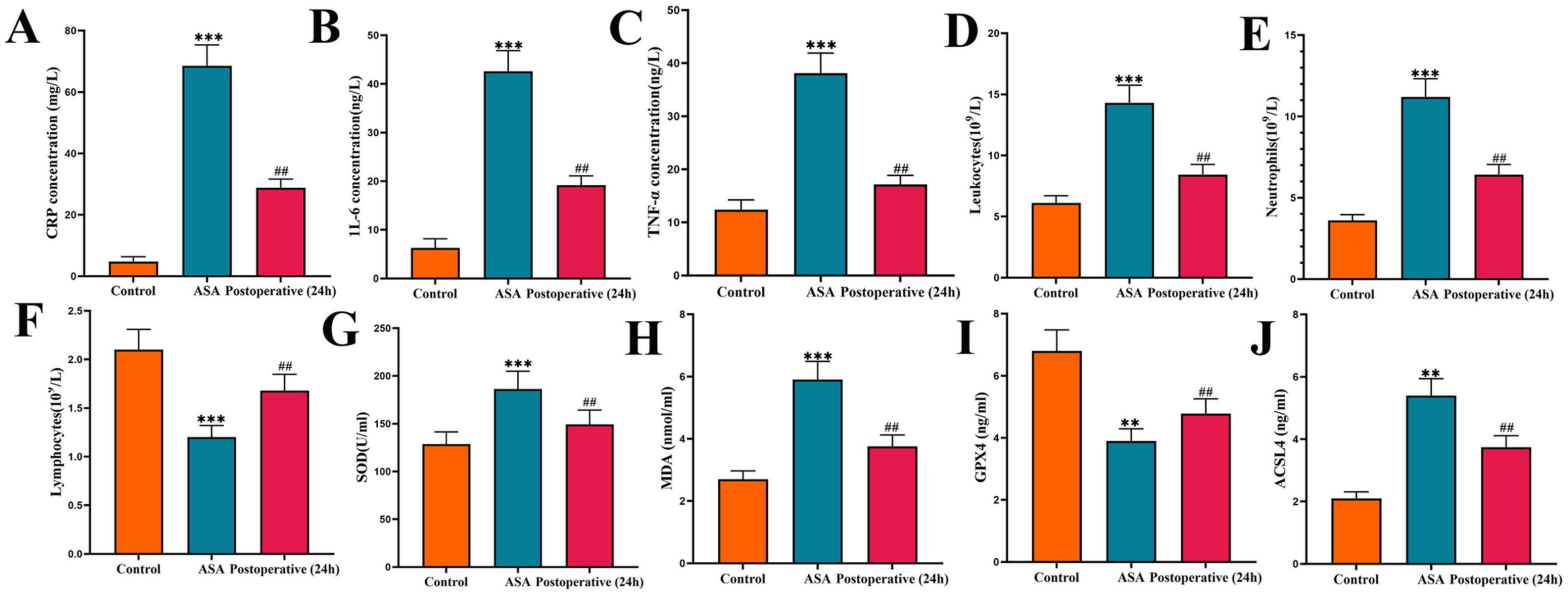
Intergroup comparison of inflammatory markers, blood cell counts, and oxidative stress-related markers before and after surgery. **(A)** Comparison of CRP levels. **(B)** Comparison of IL-6 levels. **(C)** Comparison of TNF-α levels. **(D)** Comparison of white blood cell counts. **(E)** Comparison of neutrophil counts. **(F)** Comparison of lymphocyte counts. **(G)** Comparison of SOD levels. **(H)** Comparison of MDA levels. **(I)** Comparison of GPX4 levels. **(J)** Comparison of ACSL4 levels. ***p < 0.001, **p < 0.01 compared with the control group; ##*p* < 0.01, compared with the ASA group. CRP, C-reactive protein; IL-6, interleukin-6; MDA, malondialdehyde; SOD, superoxide dismutase; TNF-α, tumor necrosis factor-α; GPX4, glutathione peroxidase 4; ACSL4, acyl-CoA synthase long chain family member 4.

To assess the systemic inflammatory burden in patients with acute suppurative appendicitis (ASA), this study used ELISA to detect the expression of serum CRP, IL-6, and TNF-α, and analyzed peripheral blood leukocytes (WBCs) and their classification using blood smear examination. Results showed that, compared with the control group, preoperative serum CRP, IL-6, and TNF-α levels were significantly elevated in ASA patients, peripheral blood WBC and neutrophil counts were significantly increased, while lymphocyte counts were significantly decreased. All differences between groups were statistically significant (*p* < 0.05), indicating a significant systemic inflammatory response in ASA patients (Figures 4A–F). Further analysis of inflammatory marker changes 24 h postoperatively revealed that, compared with preoperative levels, serum CRP, IL-6, and TNF-α levels showed a significant decreasing trend 24 h postoperatively, peripheral blood WBC and neutrophil counts also decreased accordingly, while lymphocyte counts increased compared with preoperative levels. These differences were statistically significant (*p* < 0.05). However, compared with the control group, the above-mentioned inflammatory markers remained at relatively high levels 24 h postoperatively, suggesting that although the inflammatory response in patients had partially subsided in the early postoperative period, it had not yet fully returned to normal. Further detection of oxidative stress and ferroptosis-related markers revealed that, compared with the control group, serum SOD and MDA levels in ASA patients were significantly elevated before surgery (*p* < 0.001), indicating a significantly enhanced level of oxidative stress. Simultaneously, ferroptosis-related molecular analysis showed that GPX4 expression was significantly downregulated and ACSL4 expression was significantly upregulated in serum of ASA patients before surgery (*p* < 0.01), indicating a significant change in the expression profile of ferroptosis-related molecules in ASA patients. Compared with preoperative levels, serum MDA levels in ASA patients decreased significantly 24 h postoperatively, while SOD levels showed a recovery trend. Meanwhile, GPX4 expression increased compared with preoperative levels, while ACSL4 expression decreased, with statistically significant differences (both *p* < 0.05). However, compared with the control group, these markers had not yet fully returned to normal levels (Figures 4G–J). The meta-analysis results showed that ASA patients not only exhibited a significant systemic inflammatory response, but also increased levels of oxidative stress and abnormal expression of ferroptosis-related molecules. These changes provide a biological basis for further exploring the potential relationship between inflammatory burden and hemodynamic parameters of SMA.

### 3.4 Relationship between SMA-PSV and inflammatory factors and oxidative stress markers

Table 2 Pearson correlation analysis showed that SMA-PSV had significant positive correlations with multiple inflammatory markers and oxidative stress indicators. Specifically, SMA-PSV was significantly positively correlated with CRP (*r* = 0.554, *p* < 0.001), IL-6 (*r* = 0.603, *p* < 0.001), and TNF-α (*r* = 0.714, *p* < 0.001). Furthermore, SMA-PSV also showed significant positive correlations with white blood cell count (*r* = 0.688, *p* < 0.001), neutrophil count (*r* = 0.722, *p* < 0.001), and lymphocyte count (*r* = 0.681, *p* < 0.001). Regarding oxidative stress indicators, SMA-PSV showed significant positive correlations with both superoxide dismutase (SOD) (*r* = 0.645, *p* < 0.001) and malondialdehyde (MDA) (*r* = 0.493, *p* < 0.001). Furthermore, SMA-PSV also exhibited significant positive correlations with the ferroptosis-related biomarkers GPX4 (*r* = 0.561, *p* < 0.001) and ACSL4 (*r* = 0.433, *p* < 0.001). These results suggest that SMA-PSV may serve as an important hemodynamic parameter for assessing the body’s inflammatory response and oxidative stress status, providing potential predictive value for clinically evaluating patients’ inflammation and oxidative stress levels.

**Table 2:** Correlation between SMA-PSV and various inflammatory markers and oxidative stress indices.

Factor	*r*	*p*
CRP (mg/L)	0.554	<0.001
IL-6 (ng/L)	0.603	<0.001
TNF-α (ng/L)	0.714	<0.001
Leukocyte (×10^9^/L)	0.688	<0.001
Neutrophils (×10^9^/L)	0.722	<0.001
Lymphocytes (×10^9^/L)	0.681	<0.001
SOD (U/mL)	0.645	<0.001
MDA (μmol/mL)	0.493	<0.001
GPX4 (ng/L)	0.561	<0.001
ACSL4 (ng/L)	0.433	<0.001

*p* < 0.05 indicates a significant difference.

### 3.5 Changes in the expression of inflammatory factors and vasoactive factors in appendiceal tissue after ASA surgery

**Figure 5:**
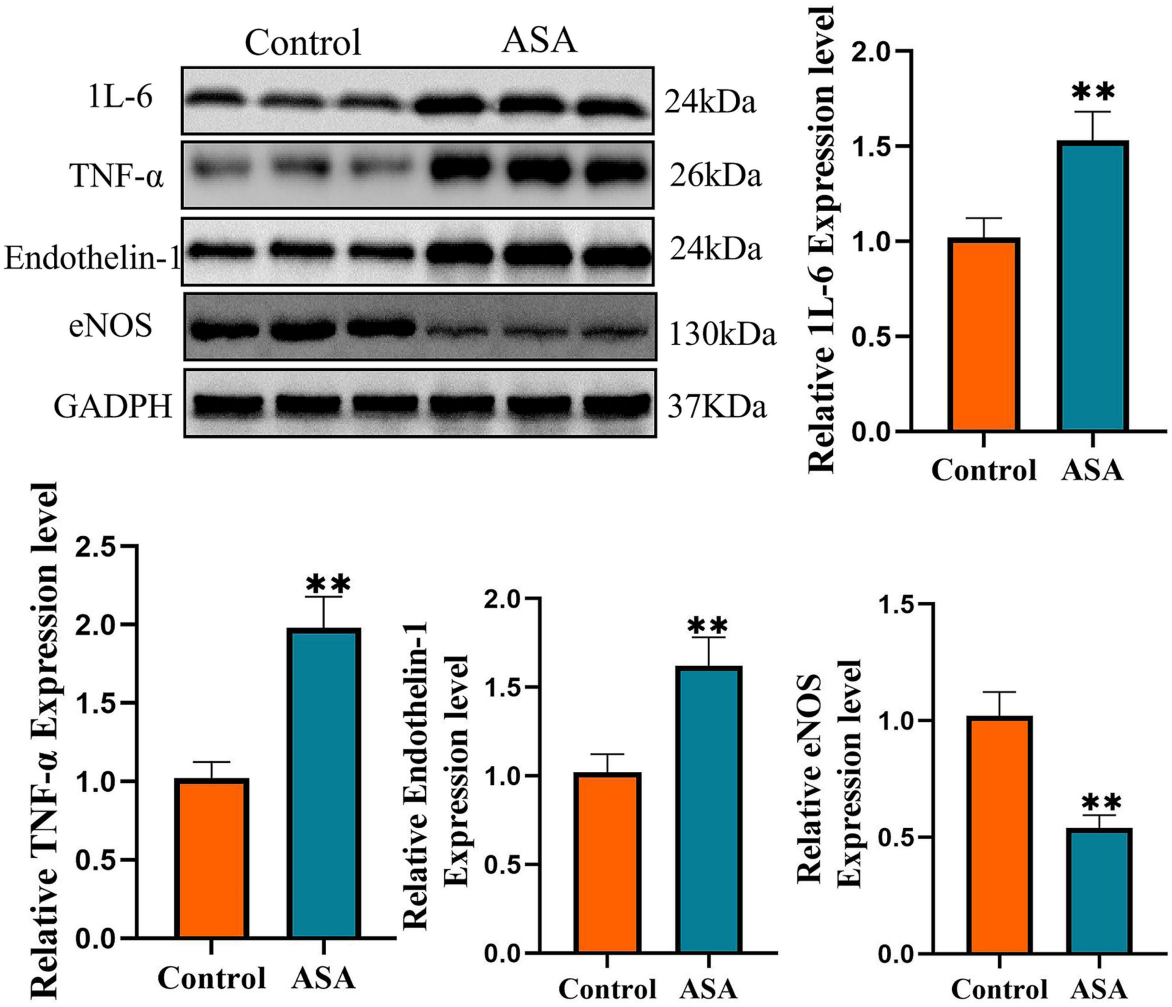
Changes in the expression of IL-6, TNF-α, endothelin-1, and eNOS in appendiceal tissue after ASA surgery. The expression levels of IL-6, TNF-α, and endothelin-1 in the ASA group were significantly higher than those in the control group (*p* < 0.01), while the expression level of eNOS was significantly lower (*p* < 0.01). **p < 0.01. All data are the mean ± standard deviation of three replicates, *n* = 3.

Given the significantly increased blood flow resistance in SMA patients with ASA, we further analyzed the expression of local vasoactive factors in the appendix. Western blot results showed that the expression of inflammatory factors and vasoactive proteins in the appendix tissue after ASA surgery was significantly different from that in non-inflammatory appendix tissue. Specifically, the expression of IL-6 and TNF-α proteins was significantly upregulated, indicating an active local inflammatory response. In addition, the expression of the vasoactive factor ET-1 was significantly increased, while the expression of eNOS was significantly decreased, suggesting impaired endothelial vasodilatory function (Figure 4). These changes are consistent with the trend of increased hemodynamic parameters, suggesting that local inflammation may play an important role in the mechanism of SMA blood flow changes by regulating the expression of vasoactive factors (see Figure 5).

## 4 Discussion

The appendix develops as an outpouching at the junction between the small intestine and the colon around the fifth week of intrauterine growth (17). Its anatomical position varies and can be retrocecal, pelvic, subcecal, preileal, postileal, or ectopic (18). Given this variability, the location of the appendix tip should be carefully considered in clinical assessments. Structurally, the human appendix measures approximately 10 cm in length and 7–8 mm in diameter, with a wall thickness of 1–3 mm (17, 19). Appendicitis commonly results from luminal obstruction, which facilitates bacterial invasion of the appendix wall. As intraluminal pressure increases, it can lead to venous congestion, arterial insufficiency, ischemia, necrosis, and ultimately perforation. Histologically, acute appendicitis (AA) is classified into different subtypes, including simple, purulent, perforated, and gangrenous appendicitis (20).

Acute suppurative appendicitis (ASA) is a severe form of appendicitis characterized by involvement of the entire appendiceal wall, significant swelling of the appendix, and an elevated outer appendiceal plasma membrane (21). If left untreated, ASA may progress to gangrenous appendicitis, which has a poor prognosis. The diagnosis of ASA is primarily based on clinical symptoms and laboratory findings. Among imaging modalities, ultrasound is often the preferred initial examination due to its non-radiative nature, lack of sedation requirements, and relatively low cost (13, 22, 23). Studies have also suggested that repeat ultrasound evaluations can help distinguish appendicitis from other conditions when CT findings are inconclusive (24).

However, ultrasound-based assessment of the appendix presents several challenges. First, the variable anatomical location of the appendix tip makes visualization difficult. Second, obesity and abdominal distension can hinder ultrasound penetration. Third, severe inflammation in the appendix tail can lead to significant gas accumulation in the surrounding intestinal lumen, which further obscures imaging. Additionally, the experience and technique of the ultrasonographer can impact the accuracy of the examination. Due to these limitations, the risk of missed or misdiagnosed cases of ASA remains high. Thus, exploring alternative diagnostic methods is essential for improving the detection and management of ASA.

In this study, we aimed to enhance ASA diagnosis by integrating superior mesenteric artery (SMA) hemodynamic analysis with traditional two-dimensional ultrasound examination. By assessing SMA blood flow parameters, we sought to evaluate the severity of appendicitis and facilitate a more comprehensive diagnosis of ASA. This approach may help reduce the diagnostic errors associated with conventional appendix-focused ultrasound examinations.

Recent studies have highlighted the clinical significance of SMA assessment due to its accessibility via ultrasound and its involvement in various abdominal pathologies (25). The SMA, a major visceral branch of the abdominal aorta, has a long main artery and a broad blood supply range, making it relevant to conditions beyond appendicitis, including diabetes, atherosclerosis, and inflammatory bowel disease (26, 27). Several studies have demonstrated that hemodynamic alterations in the SMA can serve as valuable diagnostic indicators (28–30). For instance, Arienti et al. and Alvarez et al. reported increased peak systolic velocity (PSV), end-diastolic velocity (EDV), and blood flow volume, alongside decreased resistance index (RI) and pulsatility index (PI), in patients with inflammatory bowel disease and celiac disease (31, 32). Similarly, Erden et al. (33) observed higher PSV and EDV values and lower RI values in patients with various small-bowel diseases. Berat et al. found significant increases in PSV, EDV, and blood flow volume in patients with mesenteric panniculitis, accompanied by corresponding decreases in PI and RI.

Furthermore, SMA hemodynamics vary with age, vascular structure, and physiological conditions. Juliana et al. demonstrated that SMA blood flow is significantly lower in children compared to adolescents and is correlated with body surface area (34). These findings suggest that SMA blood flow dynamics are influenced by multiple factors, necessitating further research into age-specific reference values for diagnostic applications.

By integrating SMA hemodynamic parameters into ultrasound-based assessments, our study provides a novel approach to improving ASA diagnosis. This method has the potential to enhance diagnostic accuracy, reduce reliance on CT imaging, and facilitate early intervention, ultimately improving patient outcomes. Future research should focus on validating SMA-based diagnostic criteria across diverse patient populations and exploring its utility in distinguishing ASA from other acute abdominal conditions.

Our study focused on the age group most commonly affected by acute appendicitis, specifically individuals between 20 and 30 years old. The incidence of appendicitis in this population was significantly higher than in other age groups, highlighting the importance of early and accurate diagnosis in this demographic.

This study aimed to investigate the relationship between superior mesenteric artery (SMA) hemodynamic changes and acute suppurative appendicitis (ASA). Compared with the control group, patients with ASA exhibited significantly different peak systolic velocity (PSV), resistance index (RI), and systolic-to-diastolic ratio (S/D), indicating a clear hemodynamic response to acute suppurative inflammation of the appendix. Our findings suggest that inflammation-induced changes in the appendiceal artery lead to increased blood flow, which in turn elevates PSV. In contrast, end-diastolic velocity (EDV) showed minimal differences between the two groups, likely because EDV variations are primarily associated with intrinsic vascular abnormalities such as severe stenosis or occlusion. Given that increased blood flow alone has limited influence on EDV, PSV serves as a more reliable indicator of hemodynamic changes in ASA.

To further evaluate the diagnostic potential of SMA hemodynamics, we performed receiver operating characteristic (ROC) curve analysis to establish a threshold for PSV. The area under the curve (AUC) for PSV in diagnosing ASA was 0.89, suggesting moderate diagnostic accuracy. The optimal cut-off value for PSV was determined to be 157.5 cm/s. Therefore, when a patient is suspected of having ASA, an SMA hemodynamic assessment should be performed, and an elevated PSV, particularly above 157.5 cm/s, may strongly indicate the presence of ASA.

SMA vascular structural abnormalities (such as atherosclerotic stenosis, occlusion, and aneurysmal dilatation) can significantly affect hemodynamic parameters. To minimize interference, this study excluded patients with a history of SMA disease, ensuring that the included subjects had relatively normal SMA anatomy. The control group consisted of healthy individuals without cardiovascular, metabolic, or chronic vascular diseases. Systemic inflammation can lead to vasodilation and elevated SMA-PSV through pro-inflammatory factors such as IL-6 and TNF-α. This study only included patients with suppurative appendicitis confined to the appendix (excluding perforation and surrounding abscesses), where systemic inflammation was controllable. SMA-PSV was significantly positively correlated with CRP (*r* = 0.554), IL-6 (*r* = 0.603), TNF-α (*r* = 0.714), white blood cell count (*r* = 0.688), and neutrophil count (*r* = 0.722) (*p* < 0.001), suggesting that hemodynamic changes are synchronous with local inflammatory burden and have pathophysiological specificity to appendicitis.

To our knowledge, few studies have examined the role of SMA hemodynamics in ASA diagnosis. The most significant contribution of this study is its potential clinical application: when ASA is suspected, but direct visualization of the appendix via ultrasound is challenging due to anatomical variations or patient-related factors, evaluating SMA hemodynamic changes can serve as an alternative diagnostic approach. This method provides an additional clinical basis for diagnosing ASA, potentially improving early detection and management.

Despite its strengths, our study has certain limitations. First, we did not analyze the influence of factors such as gender, height, and body mass index on SMA hemodynamics, which could introduce variability in the findings. Second, due to the limited existing literature on SMA hemodynamics in ASA, further research is required to validate these findings and explore their broader clinical applicability.

This study systematically evaluated the hemodynamic characteristics of the superior mesenteric artery (SMA) in patients with acute suppurative appendicitis (ASA). Results showed significantly elevated PSV, RI, and S/D. PSV demonstrated good diagnostic efficacy in ASA (AUC = 0.89) and was positively correlated with inflammation, oxidative stress, and ferroptosis-related molecules. SMA hemodynamic assessment can serve as a non-invasive and reliable auxiliary diagnostic tool, especially when appendiceal ultrasound imaging is limited, aiding in the early identification and accurate diagnosis of ASA and providing quantitative reference for clinical decision-making.

## Data Availability

The raw data supporting the conclusions of this article will be made available by the authors, without undue reservation.

## References

[1] Borruel Nacenta S Ibanez Sanz L Sanz Lucas R Depetris MA Martinez CE. Update on acute appendicitis: typical and untypical findings. Radiologia (Engl Ed). (2023) 65:S81–91. doi: 10.1016/j.rxeng.2022.09.010, 37024234

[2] Snyder MJ Guthrie M Cagle S. Acute appendicitis: efficient diagnosis and management. Am Fam Physician. (2018) 98:25–33. 30215950

[3] Di Saverio S Podda M De Simone B Ceresoli M Augustin G Gori A . Diagnosis and treatment of acute appendicitis: 2020 update of the WSES Jerusalem guidelines. World J Emerg Surg. (2020) 15:27. doi: 10.1186/s13017-020-00306-3, 32295644 PMC7386163

[4] Guo Y Ye D Yang G Liu G Cui X Tan S . Cluster of acute appendicitis among high school Tibetan students in Nanchang, China: investigation, control, and prevention. Front Public Health. (2022) 10:889793. doi: 10.3389/fpubh.2022.889793, 35493398 PMC9051332

[5] Bhangu A Søreide K Di Saverio S Assarsson JH Drake FT. Acute appendicitis: modern understanding of pathogenesis, diagnosis, and management. Lancet. (2015) 386:1278–87. doi: 10.1016/s0140-6736(15)00275-5, 26460662

[6] Casanova-Díaz AS. Acute appendicitis. Bol Asoc Med P R. (1989) 81:167–9. 2660839

[7] Saidi HS Adwok JA. Acute appendicitis: an overview. East Afr Med J. (2000) 77:152–6. doi: 10.4314/eamj.v77i3.46612, 12858891

[8] Man Y Li S Yu Z. Septic shock caused by acute appendicitis complicated with abscess formation within mesoappendix: a case report. Int J Surg Case Rep. (2020) 76:186–9. doi: 10.1016/j.ijscr.2020.09.159, 33038845 PMC7550827

[9] Moris D Paulson EK Pappas TN. Diagnosis and management of acute appendicitis in adults: a review. JAMA. (2021) 326:2299–311. doi: 10.1001/jama.2021.20502, 34905026

[10] Ferris M Quan S Kaplan BS Molodecky N Ball CG Chernoff GW . The global incidence of appendicitis. Ann Surg. (2017) 266:237–41. doi: 10.1097/sla.0000000000002188 28288060

[11] Gallo G Podda M Goglia M Di Saverio S. "Acute appendicitis". In: Coccolini F editor. Textbook of Emergency General Surgery. (Milan: Springer Cham) (2023). p. 983–1000.

[12] Kim M Kim SJ Cho HJ. Effect of surgical timing and outcomes for appendicitis severity. Ann Surg Treat Res. (2016) 91:85–9. doi: 10.4174/astr.2016.91.2.85, 27478814 PMC4961891

[13] Alerhand S Meltzer J Tay ET. Evaluating the risk of appendiceal perforation when using ultrasound as the initial diagnostic imaging modality in children with suspected appendicitis. Ultrasound. (2017) 25:166–72. doi: 10.1177/1742271X16689693, 29410692 PMC5794046

[14] Acu B Güven ME Kaptan MA Öztunalı Ç Gökçe E Beyhan M . Duplex Doppler sonographic assessment of the superior mesenteric artery in patients with mesenteric panniculitis. J Ultrasound Med. (2018) 37:165–72. doi: 10.1002/jum.14314, 28731594

[15] Siğirci A Baysal T Kutlu R Aladağ M Saraç K Harputluoğlu H. Doppler sonography of the inferior and superior mesenteric arteries in ulcerative colitis. J Clin Ultrasound. (2001) 29:130–9. doi: 10.1002/1097-0096(200103/04)29:3<130::aid-jcu1012>3.0.co;2-x 11329155

[16] Yekeler E Danalioglu A Movasseghi B Yilmaz S Karaca C Kaymakoglu S . Crohn disease activity evaluated by Doppler ultrasonography of the superior mesenteric artery and the affected small-bowel segments. J Ultrasound Med. (2005) 24:59–65. doi: 10.7863/jum.2005.24.1.59, 15615929

[17] Agrawal M Allin KH Mehandru S Faith J Jess T Colombel JF. The appendix and ulcerative colitis - an unsolved connection. Nat Rev Gastroenterol Hepatol. (2023) 20:615–24. doi: 10.1038/s41575-023-00774-3, 37081213 PMC10527463

[18] Penny SM. Imaging the vermiform appendix. Radiol Technol. (2018) 89:571–90. 30420527

[19] Smith HF Fisher RE Everett ML Thomas AD Bollinger RR Parker W. Comparative anatomy and phylogenetic distribution of the mammalian cecal appendix. J Evol Biol. (2009) 22:1984–99. doi: 10.1111/j.1420-9101.2009.01809.x, 19678866

[20] Huerta S. Diagnosis and management of acute appendicitis. JAMA. (2022) 327:1183–4. doi: 10.1001/jama.2022.1265 35315897

[21] Zhang N Li Y Zhou R. Comparison of single-person laparoscopic appendectomy using a novel brace-assisted camera holding system and conventional laparoscopic appendectomy: a neural network algorithm analysis. Contrast Media Mol Imaging. (2022) 2022:5915670. doi: 10.1155/2022/5915670, 36349334 PMC9630036

[22] Xiao Y Jian G Zhong Y Chen J Ye J Chen Y . Ultrasound and clinical features for differential diagnosis of low-grade appendiceal mucinous neoplasm and acute suppurative appendicitis. Med Ultrason. (2024) 26:348–55. doi: 10.11152/mu-4412, 39078992

[23] Eng KA Abadeh A Ligocki C Lee YK Moineddin R Adams-Webber T . Acute appendicitis: a Meta-analysis of the diagnostic accuracy of US, CT, and MRI as second-line imaging tests after an initial US. Radiology. (2018) 288:717–27. doi: 10.1148/radiol.2018180318, 29916776

[24] Kim MS Kwon HJ Kang KA do IG Park HJ Kim EY . Diagnostic performance and useful findings of ultrasound re-evaluation for patients with equivocal CT features of acute appendicitis. Br J Radiol. (2018) 91:20170529. doi: 10.1259/bjr.20170529, 29099612 PMC5965797

[25] Taylor GA. Blood flow in the superior mesenteric artery: estimation with Doppler US. Radiology. (1990) 174:15–6. doi: 10.1148/radiology.174.1.2403677, 2403677

[26] Mathenge N Osiro S Rodriguez II Salib C Tubbs RS Loukas M. Superior mesenteric artery syndrome and its associated gastrointestinal implications. Clin Anat. (2014) 27:1244–52. doi: 10.1002/ca.22249, 23959808

[27] Zhao YE Wang ZJ Zhou CS Zhu FP Zhang LJ Lu GM. Multidetector computed tomography of superior mesenteric artery: anatomy and pathologies. Can Assoc Radiol J. (2014) 65:267–74. doi: 10.1016/j.carj.2013.10.001, 24874500

[28] Meneghini LF Hogan AR Selvaggi G. Superior mesenteric artery syndrome in type 1 diabetes masquerading as gastroparesis. Diabetes Care. (2008) 31:1983–4. doi: 10.2337/dc08-0544, 18628573 PMC2551639

[29] Hirai T Kitada M Hayashi Y Monno I Takagaki Y Shimada K . Case report of superior mesenteric artery syndrome that developed in a lean type 2 diabetes patient and was associated with rapid body weight loss after sodium-glucose cotransporter 2 inhibitor administration. J Diabetes Investig. (2020) 11:1359–62. doi: 10.1111/jdi.13228, 32020751 PMC7477529

[30] Lin TC Wright CM Criqui MH Allison MA. Superior mesenteric artery calcification is associated with cardiovascular risk factors, systemic calcified atherosclerosis, and increased mortality. J Vasc Surg. (2018) 67:1484–90. doi: 10.1016/j.jvs.2017.08.081, 29103930 PMC6696994

[31] Arienti V Califano C Brusco G Boriani L Biagi F Giulia Sama M . Doppler ultrasonographic evaluation of splanchnic blood flow in coeliac disease. Gut. (1996) 39:369–73. doi: 10.1136/gut.39.3.369, 8949639 PMC1383341

[32] Alvarez D Vazquez H Bai JC Mastai R Flores D Boerr L. Superior mesenteric artery blood flow in celiac disease. Dig Dis Sci. (1993) 38:1175–82. doi: 10.1007/bf01296064 8325179

[33] Erden A Cumhur T Olçer T. Superior mesenteric artery blood flow in patients with small bowel diseases: evaluation with duplex Doppler sonography. J Clin Ultrasound. (1998) 26:37–41. doi: 10.1002/(sici)1097-0096(199801)26:1<37::aid-jcu8>3.0.co;2-k, 9475207

[34] Eloi JC Epifanio M Spolidoro JV Camargo P Krebs J Mizerkowski MD . Doppler US measurement of the superior mesenteric artery blood flow in children and adolescents. Pediatr Radiol. (2012) 42:1465–70. doi: 10.1007/s00247-012-2484-1, 22956178

